# Circadian Gene NPAS2 Relieves Hypertrophic Scar Formation via CDC25A‐Mediated Fibroblasts Activity

**DOI:** 10.1111/jcmm.70643

**Published:** 2025-06-23

**Authors:** Pei Wei, Yongqiang Xiao, Zhaorong Xu, Xiaodong Chen, Qiong Jiang, Yu Fu, Jianji Yan, Zhaohong Chen, Pengfei Luo, Huazhen Liu

**Affiliations:** ^1^ Burn & Wound Repair Department Fujian Medical University Union Hospital Fuzhou Fujian China; ^2^ Fujian Burn Institute Fujian Medical University Union Hospital Fuzhou Fujian China; ^3^ Fujian Burn Medical Center Fujian Medical University Union Hospital Fuzhou Fujian China; ^4^ Fujian Provincial Key Laboratory of Burn and Trauma Fujian Medical University Union Hospital Fuzhou Fujian China; ^5^ Plastic Surgery Department of Shanghai Ninth People's Hospital Shanghai Ninth People's Hospital Shanghai Jiao Tong University School of Medicine Shanghai China; ^6^ Department of Burn Surgery, Changhai Hospital Naval Medical University Shanghai China; ^7^ Research Unit of Key Techniques for Treatment of Burns and Combined Burns and Trauma Injury Chinese Academy of Medical Sciences, Changhai Hospital Shanghai China

**Keywords:** CDC25A, circadian clock, fibrosis, hypertrophic scar, NPAS2

## Abstract

Neuronal PAS domain protein 2 (NPAS2) is critical in tissue fibrosis. Hypertrophic scars (HTS), a form of skin fibrosis, are characterised by excessive myofibroblast proliferation and abnormal extracellular matrix (ECM) deposition. However, whether NPAS2 contributes to skin fibrosis and the development of HTS remains unclear. In this study, the expression of NPAS2 between normal skin and hypertrophic scars (HTS) was assessed using RT‐qPCR and immunohistochemistry (IHC). Human dermal fibroblasts (HDFs) and HTS‐derived fibroblasts (HTS‐Fs) were isolated from normal skin and HTS, respectively. NPAS2 was knocked down in HTS‐Fs and overexpressed in HDFs via gene transfection. Cell proliferation and migration of transfected HTS‐Fs and HDFs were analysed using flow cytometry, CCK‐8 and transwell assays. The expressions of NPAS2, CLOCK, BMAL1, COL I, COL III, α‐SMA and CDC25A were evaluated by western blotting and RT‐qPCR. Dual‐luciferase reporter assays and chromatin immunoprecipitation (ChIP) identified the regulatory effect of NPAS2 on CDC25A. In vivo, an 8 × 8 mm full‐thickness skin defect was created on the tail of SD rats, with viral particles (1 × 107) of r‐plenR‐sh‐NPAS2 or r‐plenR‐NPAS2‐NC injected subcutaneously at the wound edges weekly. Tissue samples, histopathological analyses and photographs were taken until the wound healed completely. The results indicated that NPAS2 was significantly upregulated in HTS. The proliferation, migration, and expression of COL I, COL III, and α‐SMA were higher in HDFs overexpressing NPAS2 than those of HDFs themselves. In contrast, the behaviours mentioned above of HTS‐Fs knocking down NPAS2 were lower than that of HTS‐Fs. Mechanistically, the migration and proliferation promoting effect of NPAS2 was mediated by the binding of NPAS2 to the E‐like‐box of CDC25A. In vivo, compared with the r‐plenR‐NPAS2‐NC group, the re‐epithelialised regions of r‐plenR‐sh‐NPAS2 were pink, flat and as large as the initial wound. In addition, their dermal structures were similar to skin and possessed loose and regular collagen arrangement which was parallel to the epidermis. Take together, these findings suggested that compared with HDFs, NPAS2 was upregulated in HTS‐Fs. NPAS2 promoted the activation of HDFs, which is characterised by stronger proliferation and migration and the higher level of α‐SMA, COL I and COL III. In which, the proliferation and migration effects of NPAS2 were mediated by CDC25A. Furthermore, NPAS2 knocked down in rat tail wounds inhibited the HTS formation. Therefore, NPAS2 may serve as a potential therapeutic target for HTS in the future.

## 1 Introduction

Hypertrophic scar (HTS) arises from traumatic or surgical injuries and the subsequent aberrant form of wound healing, which is characterised by excessive proliferation of myofibroblasts and pathological deposit of extracellular matrix (ECM) [1]. It is reported that the prevalence of HTS ranges from 32% to 72% [2]. Patients suffering from HTS may result in contracture deformities, which lead to significant disfigurement or disability and prevent patients from integrating into society to approach life [3, 4]. Currently, the prevention of HTS primarily involves optimising the wound microenvironment, expediting re‐epithelisation, and suppressing excessive activity of myofibroblasts. When HTS occurs, medication, oppression, operation, radiation and photoelectric therapy are options. Although these approaches have demonstrated certain efficacy, they still cannot completely address the occurrence and progression of HTS [5]. Interestingly, HTS development has a known racial and familial predisposition, suggesting a genetic link. Individuals with a genetic tendency for scarring are more likely to develop HTS, even from minor injuries [6, 7]. Therefore, gene therapy presents a potential treatment option targeting the genetic factors of HTS may lead to more effective therapies.

Circadian rhythms are ubiquitous at all levels of life, which are governed by a complete clock network involving a series of clock genes, such as brain and muscle arnt‐like protein 1 (BMAL1), circadian locomotor output cycles kaput (CLOCK), neuronal PAS domain protein 2 (NPAS2), Period (Per1,2,3) and Cryptochrome (Cry1,2). Circadian rhythm disorders may lead to increased susceptibility to obesity [8, 9], type II diabetes [10], high blood pressure [11] and fibrosis [12, 13, 14]. The circadian rhythm of the skin is regulated by both the central biological clock and ambient light. Abnormal skin peripheral circadian rhythm is related to the pathology and progression of skin diseases such as psoriasis and atopic dermatitis [15]. In human dermal fibroblasts (HDFs), the coordinated circadian rhythms of actin promoted cell migration and adhesion, which ultimately accelerated wound healing [16]. In HTS‐derived fibroblasts (HTS‐Fs), the BMAL1 and PER2 can modulate α‐SMA expression and cell viability, thereby inhibiting TGF‐β1‐induced differentiation of fibroblasts into myofibroblasts [17]. Meanwhile, the well‐known Smad family member 3 (Smad3) signalling pathway involved in scarring is linked to circadian rhythm and regulates cell migration and proliferation through circadian clock gene expression [18]. More importantly, disturbances in circadian rhythm could affect the synthesis and degradation of collagen, and thus interfere with the maintenance of the homeostasis of the collagen network [19]. Therefore, we hypothesised that circadian rhythm may be closely associated with HTS occurrence.

NPAS2, a homologous gene of the CLOCK, is located on chromosome 2 at 2q11.2 [20]. The combination of NPAS2 and BMAL1 forms a functional heterodimer, which subsequently modulates the expression of other downstream circadian genes and participates in the maintenance of the circadian network to preserve 24 h periodicity circadian rhythms [21, 22]. It has been reported that both CLOCK [23, 24] and BMAL1 [25, 26] are implicated in tissue fibrosis. In addition, as the homologous gene of CLOCK and the functional subunit of BMAL1, NPAS2 is involved in regulating wound healing and collagen reconstruction [27]. Interestingly, previous studies revealed that dysregulation of NPAS2 contributed to liver fibrosis [28]. However, whether it participates in skin fibrosis and affects the formation of HTS remains unknown.

In this study, we first detected the different expression of NPAS2 between normal skin and HTS tissue, and isolated HDFs and HTS‐Fs, respectively. Then, we constructed adenovirus vectors carrying NPAS2 or not, and assessed the effects of NPAS2 on the biological activity of HDFs and HTS‐Fs. Meanwhile, we developed adenovirus vectors carrying NPAS2 and explored the effects of NPAS2 on HTS in vivo experiments. Finally, we aimed to explore the beneficial effects of NPAS2 on the management of HTS.

## 2 Materials and Methods

### 2.1 Cell Isolation and Specimen Collection

HTS was diagnosed by three senior professors from the Burn and Wound Repair Department of Fujian Medical University Union Hospital. Participants suffering from HTS signed written informed consent and all procedures were approved by the Ethics Committee of Fujian Medical University Union Hospital (2021KJCX081), Fuzhou, China. HDFs and HTSFs were respectively isolated from normal skin and HTS tissues via enzyme digestion [29, 30]. Both of them were cultured in low‐sugar Dulbecco's Modified Eagle's Medium (DMEM, Gibco, USA) supplemented with 10% Fetal Bovine Serum (FBS, Gibco, USA) and 2% penicillin/streptomycin.

### 2.2 Tissue Section Preparation

Scar specimens were fixed with 4% paraformaldehyde for more than 72 h and then washed overnight in running water. Subsequently, they were successively dehydrated in a series of graded ethanol, embedded in paraffin and cut into 5 μm sheets attached to a glass slide. Finally, the slides were dried in an oven for further study.

### 2.3 Immunohistochemistry and Skin Histological Analysis

To identify the expression of NPAS2 in normal skin and HTS. Tissue slides were incubated with anti‐NPAS2 antibody (#PA5‐28298, Thermo Fisher Scientific, USA) overnight at 4°C. After that, they were incubated with biotinylated goat anti‐rabbit IgG antibody (#ab97075, Abcam, USA) in the darkroom at 37°C for 1 h, followed by incubation with horseradish peroxidase‐streptavidin and diaminobenzidine. Finally, sections were counterstained with haematoxylin, dehydrated and covered with coverslips.

### 2.4 Lentiviral Vector Construction and Transfection

Four Lentiviral vectors (GTP‐NPAS2, GTP‐NC, plenR‐sh‐NPAS2, plenR‐NC) were all constructed by Zorin Biotechnology Co. Ltd. (Shanghai, China). According to the experimental design, Lentiviral vectors were transfected into HTS‐Fs and HDFs respectively following the manufacturer's instructions. First, counting cells and a certain number of cells were planted in the six‐well plate. Second, the infection system was constructed according to the multiplicity of infection (MOI), which involves virus particles, OPTIM‐MEM (Gibco, USA) and Polybrene (4 μL/mL) without penicillin and streptomycin. Thirdly, cells were incubated with the transfection system for 12 h in 5% CO_2_ at 37°C. Finally, the transfection medium was removed for complete medium. The expression level of GFP was observed with fluorescence microscopy 24 h later to evaluate transfection efficiency. Stable cell lines were screened by guanine for further experiments. The base sequences of target genes are shown in Table 1.

**TABLE 1:** All the base sequences involved.

Gene	Base sequence	Application
h‐plenR‐sh‐NPAS2	Sense:5′CCAAGAGAGCUUCUCGAAATT‐3′ Antisense: 5′‐UUUCGAGAAGCUCUCUUGGTT‐3′	Cell transfection
h‐plenR‐NPAS2‐NC	Sense: 5′‐GUAUGACAACAGCCUCAAGTT‐3′ Antisense: 5′‐CUUGAGGCUGUUGUCAUACTT‐3′	Cell transfection
GTP‐NPAS2	NM_002518.4	Cell transfection
α‐SMA	F: 5′‐AGGTAACGAGTCAGAGCTTTGGC‐3′ R: 5′‐CTCTCTGTCCACCTTCCAGCAG‐3′	RT‐qPCR
COL I	F:5′‐GAGGGCCAAGACGAAGACATC‐3′ R: 5′CAGATCACGTCATCGCACAAC‐3′	RT‐qPCR
CDC25A	F: 5′‐TCCCTGACGAGAATAAATTCCCT‐3′ R: 5′‐TCGATGAGGTGAAAGGTGTCG‐3′	RT‐qPCR
β‐Actin	F: 5′‐GTGGCCGAGGACTTTGATTG‐3′ R: 5′‐CCTGTAACAACGCATCTCATATT‐3′	RT‐qPCR
COL III	F:5′‐CCGATGGGTTGCCAGGATCCATG‐3′ R: 5′‐GAAGGGCATTGTGCTGAACTTGCG‐3′	RT‐qPCR
NPAS2	F:5′‐ACACCCTTTCAAGACCTTGCC‐3′ R: 5′‐AGGTTCGTCAACTATGCACATTT‐3′	RT‐qPCR
E1‐box	F: 5′‐TAGCTGCCATTCGGTTGAG‐3′ R: 5′‐TCTTACCCAGGCTGTCGCAG‐3′	CHIP
E2‐box	F: 5′‐AGCTGCCACAGGTTCGGCGCTG‐3′ R: 5′‐TAGCCCCCTGGGAGCTCCTAG‐3′	CHIP
Mutent‐E2‐box	F: 5′‐ACAGTGTCATAAGCATTATGTAAAC‐3′ R: 5′‐TGCTTATGACACTGTTCCCAGTGTGT‐3′	CHIP
NPAS2	F: 5′‐ATGGATGAAGATGAGAAAGA‐3′ R: 5′‐TTATCGGGGCGGCTGCTGGA‐3′	CHIP
BMAL1	F: 5′‐ATGGCAGACCAGAGAATGGA‐3′ R: 5′‐TTACAGCGGCCATGGCAAGT‐3′	CHIP
Per1	F: 5′‐ATGAGTGGCCCCCTAGAAGG‐3′ R: 5′‐CTAGCTGGTGCAGTTTCCTG‐3′	CHIP
r‐plenR‐sh‐NPAS2	F: TTCTCCGAACGTGTCACGT R: AAGAGGCTTGCACAGTGCA	Animal treatment
r‐plenR‐NPAS2‐NC	F: GATCCGGAATTCACTTCGAGGCATAGCTTCCT GTCAGACTATGCCTCGAAGTGAATTCCTTTTTG R: AATTCAAAAAGGAATTCACTTCGAGGCATAG TCTGACAGGAAGCTATGCCTCGAAGTGAATTCCG	Animal treatment

### 2.5 RT‐qPCR

Total RNA of transfected or untransfected HDFs and HTS‐Fs was extracted and transcribed to cDNA. Then, RT‐qPCR analysis was performed, in which β‐actin served as an internal reference. Target mRNA expression level was acquired via normalisation to β‐actin. Primer sequences involved are shown in Table 1.

### 2.6 CCK‐8 Assay

Briefly, 5 × 103 cells were seeded into a 96‐well plate and cultured with low‐sugar DMEM supplemented with 2.5% FBS and 2% penicillin/streptomycin. A culture system without cells served as the blank. At 0, 12, 24, 36, 48 and 60 h later, CCK‐8 agent (10 μL/well) was added and incubated for 2 h. The absorbance was evaluated at 450 nm by a microplate reader (BioTekELx800, USA).

### 2.7 Cell Cycle Assay

Transfected or untransfected HDFs and HTS‐Fs (2 × 105–2 × 106) were collected and resuspended with 1 mL DNA staining solution and 10 μL permeabilization solution, and then incubated in the darkroom at 37°C for 30 min. After that, the distributions of the cell cycle were analysed by flow cytometer (Beckman, Los Angeles, CA, USA).

### 2.8 Western Blot Analysis

Proteins of transfected or untransfected HDFs and HTS‐Fs were collected and quantified by NCM RIPA buffer (New Cell & Molecular Biotech, Suzhou, China) and PierceTM BCA Protein Assay Kit (#23225, Thermo Fisher Scientific, USA), respectively. The primary antibodies involved are anti‐α‐SMA (#ab32575, Abcam, USA), anti‐COL I (#GTX102997, GeneTex, USA), anti‐COL III (#GTX41286, GeneTex, USA), anti‐NPAS2 (#PA5‐28298, Thermo Fisher Scientific, USA), anti‐CLOCK (#PA1‐520, Thermo Fisher Scientific, USA), anti‐BMAL1 (#PA5‐122196, Thermo Fisher Scientific, USA) and anti‐CDC25A (55031‐1‐AP, Proteintech, USA). The second antibody was horseradish peroxidase conjugated goat anti‐Rabbit IgG (#401315, CALBIOCHEM, Germany) or peroxidase conjugated goat anti‐mouse IgG (#401215, CALBIOCHEM, Germany). Protein bands were detected with maximum sensitivity substrate (#34096, Thermo Fisher Scientific, USA) and analysed by Image Pro Plus.

### 2.9 Trans‐Well Assay

Low‐serum condition not only provides sufficient nutritional support to maintain basic cellular physiological functions but also creates a nutrient‐deprivation‐like environment that suppresses proliferation [31]. So, cells were collected and resuspended in low‐sugar DMEM supplemented with 2.5% FBS and 2% penicillin/streptomycin with a density of 4 × 105/mL. Then, 200 μL cell suspension was seeded into the compartment of a transwell chamber (Corning, USA) with 8 μm pore filters. The lower 24‐well compartment was added with 600 μL complete medium. After 24 h, the cells attached to the upper surface of the filter membranes were cleaned, and migrated cells of the lower surface were fixed with formaldehyde and stained with 0.1% crystal violet for 30 min. The degree of migration was observed by an inverted microscope (Leica, Germany).

### 2.10 Dual Luciferase Reporter Assay

Two E‐like boxes of CDC25A promoter were constructed and cloned into pGL4.17 by Murui Biotechnology Co. Ltd (Suzhou, China). Meanwhile, full‐length cDNAs of Bmal1, NPAS2and CLOCK were cloned into pcDNA3.1 by Murui Biotechnology Co. Ltd (Suzhou, China) as well. HDFs were seeded into 24‐well plate and incubated to reach 70% confluence. Then, cells were co‐transfected with different combinations of pGL4.17‐CDC25A, pcDNA3.1, pcDNA3.1‐BMAL1, pcDNA3.1‐NPAS2, pcDNA3.1‐Perl and NPAS2‐siRNA. Incubated for 16 h, the transfected cells were lysed and luciferase activities were detected by Fluoroskan Ascent FL (Thermo Fisher Scientific, USA). The base sequences of target genes are shown in Table 1.

### 2.11 CHIP Assay

ChIP assay was performed strictly according to the manufacturer's protocol of the ChIP assay kit (Thermo Fisher Scientific, #26156, USA). Briefly, the cells were crosslinked with formaldehyde and lysed with Lysis Buffer. Then, nuclei were sheared by micrococcal nuclease solution and the supernatant was collected. The specific chromatin fragment was immunoprecipitated with anti‐NPAS2 antibody (ab157165, Abcam, USA) or equal amounts of Rabbit IgG (ab157165, Abcam, USA). Finally, IP was eluted and DNA extraction was amplified by ChIP‐PCR.

### 2.12 Animal Model and Treatment

The Sprague–Dawley (SD) rats (10–12 weeks old) were purchased from the experimental animal center of Fujian Medical University, Fuzhou, China. All procedures were approved by the Animal Research Committee of Fujian Medical University, Fuzhou, China. SD rats were divided randomly into two groups: r‐plenR‐sh‐NPAS2 and r‐plenR‐NPAS2‐NC. One square with sides of 8 mm full‐thickness cutaneous wound was created on the tail apart from the root 6 cm, and then the tail was rolled into a circle about 4 cm in diameter. Viral particles (1 × 10^7^) were dissolved in 200 μL low‐sugar DMEM and equally subcutaneously injected in the midpoint of the four edges for each wound once a week. Taking pictures, collecting tissue samples, and performing histopathological analyses for each wound were performed until the tail wound healed completely. The primer sequences involved are shown in Table 1.

### 2.13 Haematoxylin–Eosin and Masson's Trichrome Staining

Skin samples containing the wound beds and surrounding regions were harvested and divided into two sections along the diameter. The samples were then fixed in 4% paraformaldehyde, embedded in paraffin and cut into 5 mm sections for haematoxylin–eosin (H&E) or Masson's trichrome staining. Images were acquired with an inverted microscope (Leica DMI3000B, Germany).

### 2.14 Statistical Analysis

All data were expressed as the mean ± standard deviation (SD) and SPSS 16.0 was used for statistical analysis. Differences between two groups were analysed with Student's *t‐test*, and differences between two groups at different time points were evaluated with two‐way ANOVA. A *p* < 0.05 was considered statistically significant.

## 3 Results

### 3.1 NPAS2 Was Upregulated in HTS Tissues

**FIGURE 1:**
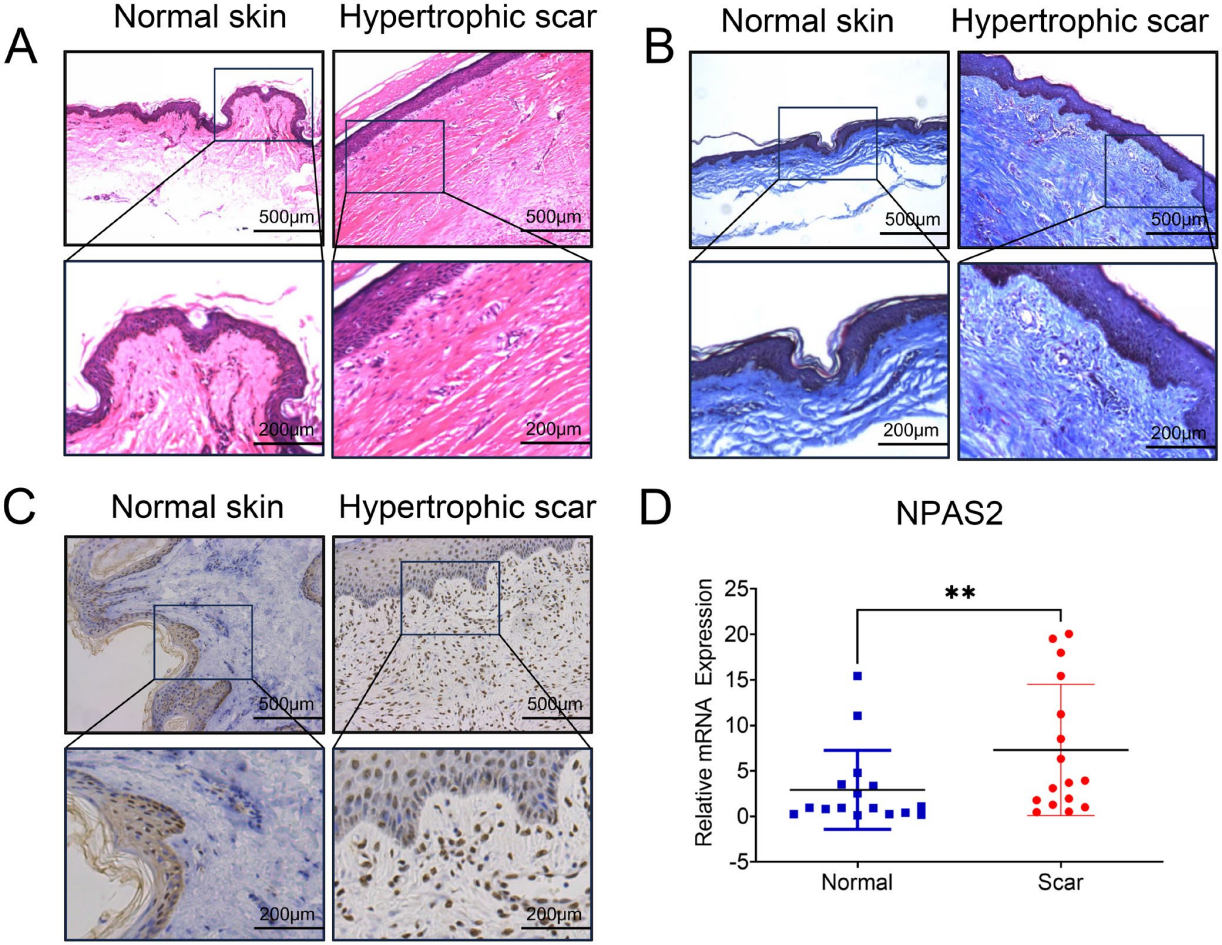
NPAS2 was upregulated in HTS tissues. (A, B) H&E staining and Masson's trichrome staining of HTS and normal skin tissues. (C) IHC staining for NPAS2 of HTS and normal skin tissues. (D) RT‐qPCR analyses for the mRNA expression level of NPAS2 in 16 paired tissues of normal skin and HTS. Scale bar, 500 and 200 μm. **p < 0.01.

Sixteen pairs of normal skin and HTS samples were collected. Just as shown in Figure 1A,B, compared with normal skin, HTS tissues possess a thicker dermal layer, in which the dermal papillae were not obvious, and the collagen arrangement was dense and irregular. Then, the results of immunohistochemistry (IHC) showed that NPAS2 was significantly upregulated in HTS tissues (Figure 1C), especially in the dermal layer. RT‐qPCR analyses provided a further support (Figure 1D) for the upregulation of NPAS2 in HTS (*p* < 0.01).

### 3.2 NPAS2 Was Upregulated in HTS‐Fs

**FIGURE 2:**
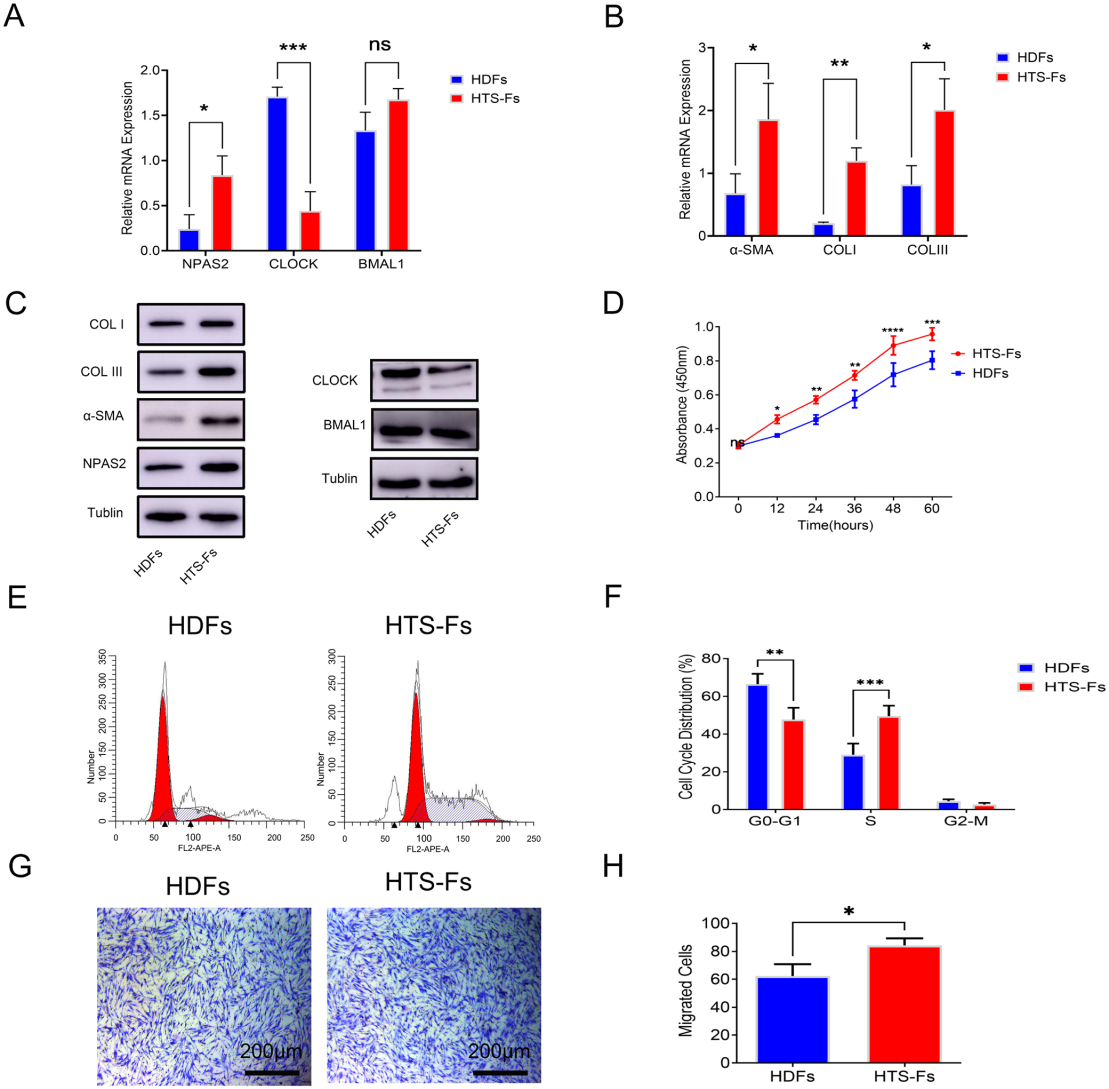
The heterogeneity in biological property of HDFs and HTS‐Fs. (A–C) RT‐qPCR and Western blot analyses for the expression levels of NPAS2, CLOCK, BMAL1, α‐SMA, COL I and COL III in HDFs and HTS‐Fs. (D) Proliferative activity of HDFs and HTS‐Fs was evaluated by CCK‐8 assay. (E, F) Cell cycle distribution of HDFs and HTS‐Fs was performed by flow cytometry. (G, H) Migration of HDFs and HTS‐Fs was assessed by transwell assay. Data shown are the mean ± SD. Scale bar, 200 μm. ns, not significant; *p < 0.05; **p < 0.01; ***p < 0.001; ****p < 0.0001.

Both RT‐qPCR and western blot analyses revealed a contrasting expression pattern between HTS‐Fs and HDFs: NPAS2, α‐SMA, COL I and COL III exhibited significant upregulation (*p* < 0.05) in HTS‐Fs, while CLOCK expression was prominently suppressed in HTS‐Fs (Figure 2A–C). CCK‐8 assays further confirmed that HTS‐Fs exhibit a much higher growth rate than HDFs (Figure 2D). Meanwhile, flow cytometry demonstrated that the proportion of cells in the G0/G1 phases was higher (*p* < 0.01), while the rate of cells in the S phase was significantly lower (*p* < 0.001) in HDFs than that in HTS‐Fs (Figure 2E,F). Moreover, Transwell assay demonstrated that HTS‐Fs exhibited a significantly higher migratory capacity compared to HDFs (Figure 2G,H).

### 3.3 NPAS2 Overexpression in HDFs Enhances Their Proliferative and Migratory Capacities

**FIGURE 3:**
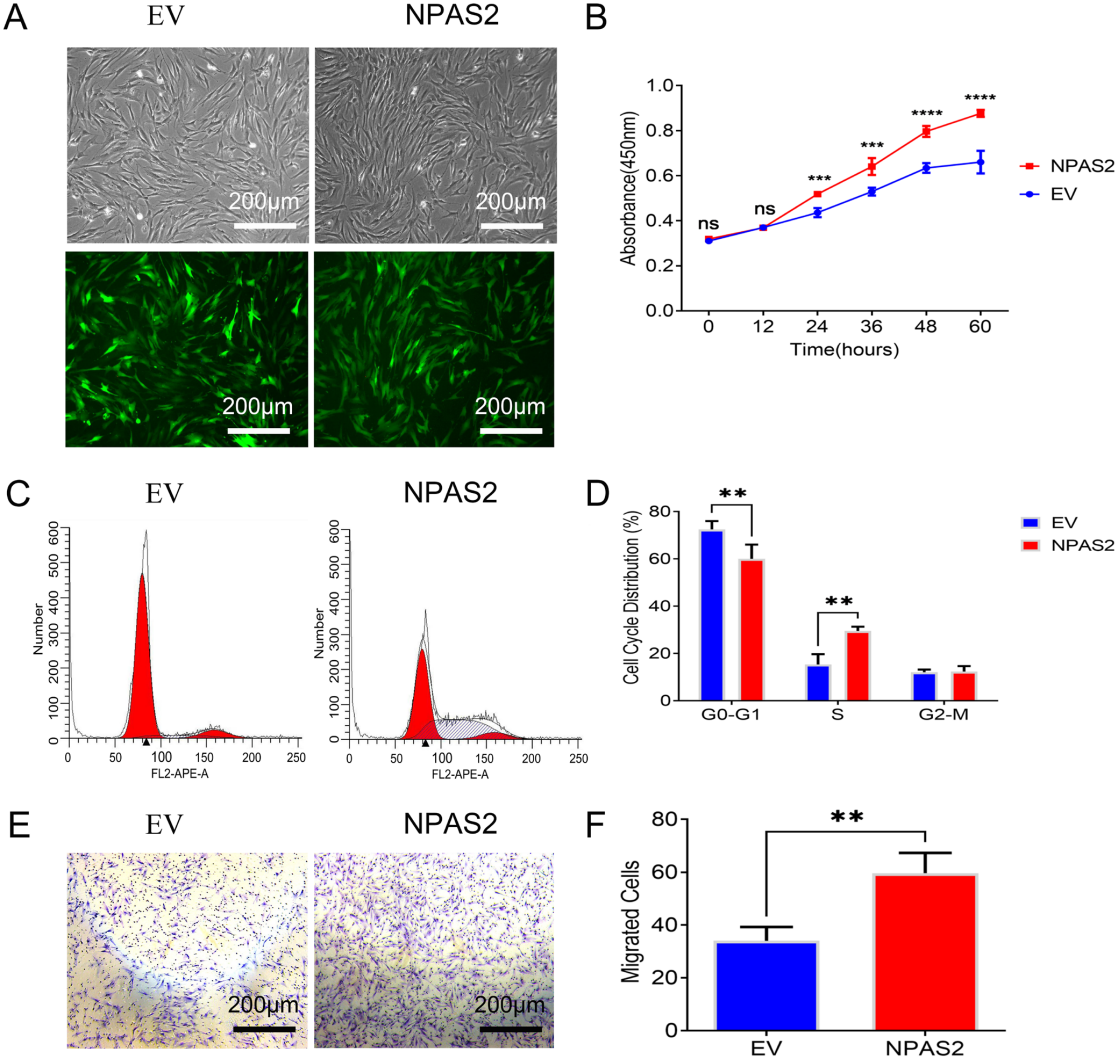
NPAS2 overexpression in HDFs promoted its biological characteristics. (A) Green fluorescent labelled lentiviral vectors overexpressed NPAS2 and control were successfully transfected into HDFs. (B) Proliferative activity of EV and NPAS2 was evaluated by CCK‐8 assay. (C, D) Cell cycle distribution of EV and NPAS2 was assessed by flow cytometry. (E) Transwell assay was applied to detect the migratory ability of EV and NPAS2. (F) Quantitative analysis of the migrated cells in (E). NPAS2, HDFs transfected with lentiviral vector overexpression NPAS2; EV, HDFs transfected with control vector. Data shown are the mean ± SD. Scale bar, 200 μm. ns, not significant; **p < 0.01; ***p < 0.001; ****p < 0.0001.

Lentiviral vectors that overexpress NPAS2 were transfected into HDFs. GFP was observed in successfully transfected HDFs by fluorescence microscopy. More than 90% of HDFs expressed green fluorescence (Figure 3A). CCK8 assay revealed that HDFs with NPAS2 overexpression possess a much higher growth rate than that of the control group (Figure 3B). In addition, flow cytometry showed that the proportion in G0/G1 phase of HDFs with NPAS2 overexpression was higher, whereas the rate of the S phase was lower than that of the control group (Figure 3C,D). Furthermore, Transwell assay demonstrated that the migration ability of HDFs with NPAS2 overexpression was stronger than that of the control group, which indicated that NPAS2 could remarkably upregulate the motility of HDFs (*p* < 0.001) (Figure 3E,F).

### 3.4 NPAS2 Knock‐Down in HTS‐Fs Inhibited Their Proliferative and Migratory Capacities

**FIGURE 4:**
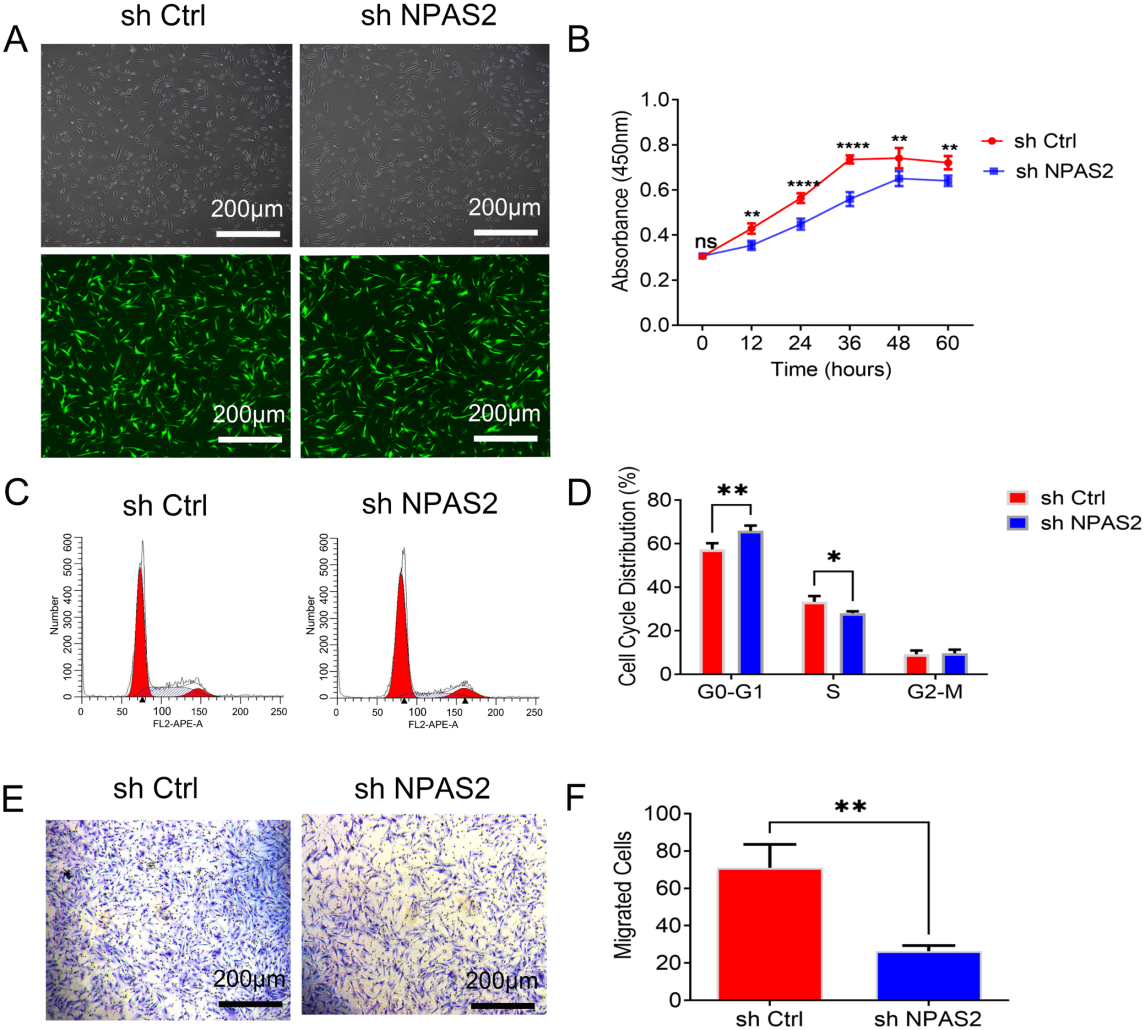
NPAS2 knocking down in HTS‐Fs inhibited its biological characteristics. (A) Green fluorescent labelled lentiviral vectors knocked down NPAS2 and control ones were successfully transfected into HSFs. (B) Proliferative activity of sh Ctrl and sh NPAS2 was evaluated by CCK‐8 assay. (C, D) Cell cycle distribution was performed for sh Ctrl and sh NPAS2 groups. (E, F) Transwell assay for the migrated cells of sh Ctrl and sh NPAS2. sh NPAS2, HTS‐Fs transfected with lentiviral vector knocking out NPAS2; sh Ctrl, HTS‐Fs transfected with control vector. Data shown are the mean ± SD. Scale bar, 200 μm. ns, not significant; *p < 0.05; **p < 0.01; ***p < 0.001; ****p < 0.0001.

Lentiviral vectors were transfected into HTS‐Fs to knock down NPAS2. GFP was observed in successfully transfected HTS‐Fs using fluorescence microscopy 24 h later (Figure 4A). CCK8 assay revealed that HTS‐Fs with NPAS2 knockdown possess a much lower growth rate than control groups (Figure 4B). Flow cytometry results further proved that the proportion in G0/G1phase of HTS‐Fs with NPAS2 knocking down was higher than that of control groups, whereas the rate of the S phase was lower (Figure 4C,D). Finally, Transwell assay demonstrated that the migration of HTS‐Fs with NPAS2 knocking down was weaker than that of the control group (*p* < 0.01) (Figure 4E,F).

### 3.5 NPAS2 Binding to the E‐Like‐Box of CDC25A

**FIGURE 5:**
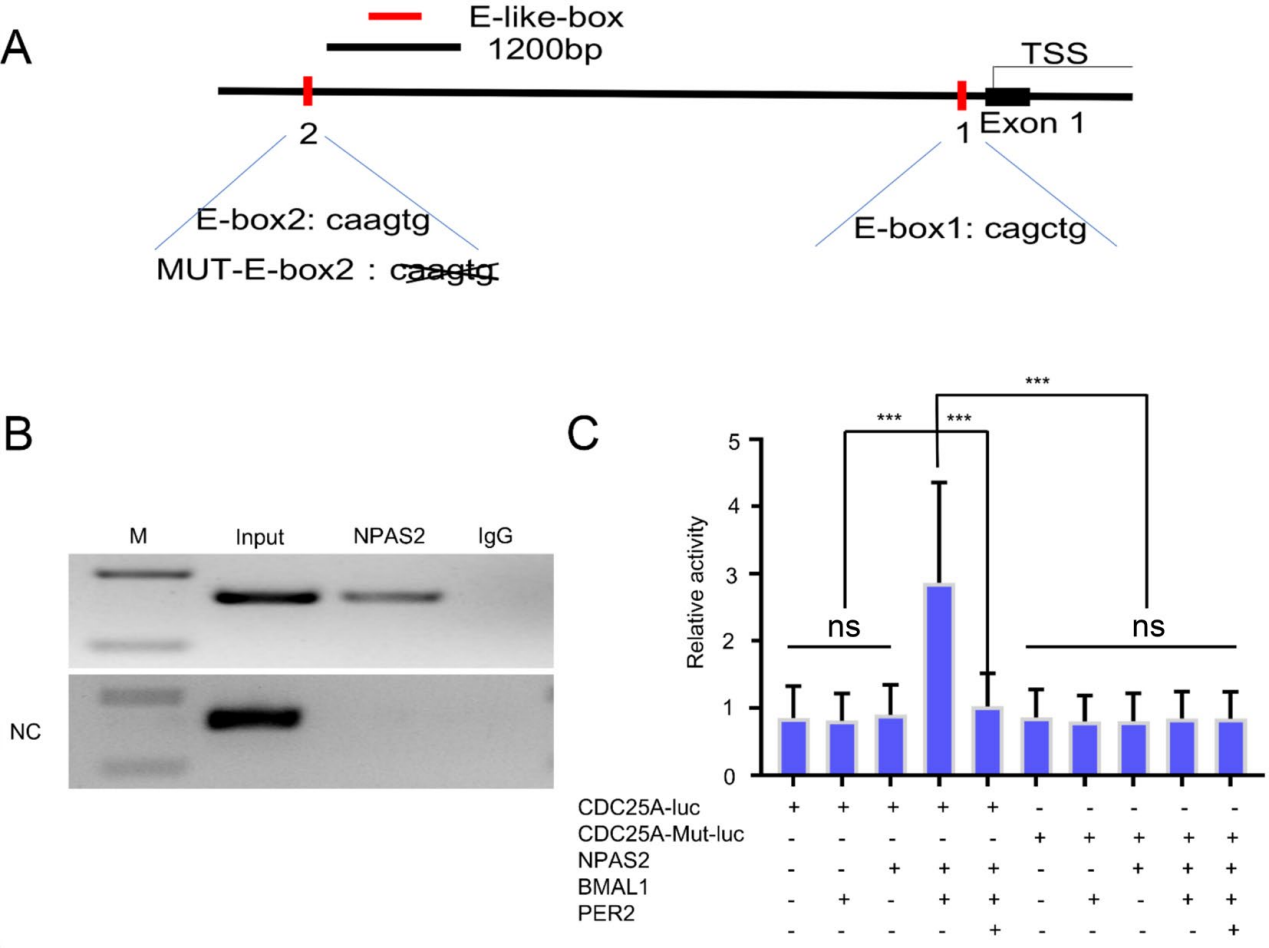
NPAS2 transcriptionally upregulated CDC25A. (A) Schematic of the E‐like boxes in the CDC25A promoter, in which NO. 1 represents −66 to −72 bp and NO. 2 represents −866 to −872 bp. (B) PCR amplification products of the CDC25A promoter sequence from ChIP DNA were detected by gel electrophoresis. Input and IgG served as positive and negative controls, respectively. In the NC (negative control) group, the CDC25A promoter lacked NO. 2 E‐like box. (C) CDC25A luciferase activity was detected by dual luciferase reporter assay. CDC25A‐luc, CDC25A‐mut‐luc, BMAL1, NPAS2 and PER2 were cotransfected into HDFs as shown above. CDC25A‐luc represents luciferase‐labelled CDC25A promoter; CDC25A‐mut‐luc represents luciferase‐labelled CDC25A promoter without NO. 2 E‐like box. Data shown are the mean ± SD. ns, not significant; ***p < 0.001.

We analysed the DNA sequence of the CDC25A promoter and distinguished two E‐like boxes, which were possible binding sites of NPAS2 (Figure 5A). CHIP assay validated that NPAS2 mainly directly binds to the second E‐likebox of the CDC25A promoter (Figure 5B). Dual luciferase reporter assay was applied to monitor the expression level of CDC25A in HDFs transfected with different transfection systems. The results showed that co‐transfection of the CDC25A promoter, Bmal1 and NPAS2 could greatly enhance the luciferase activity, whereas adding PER2 to the transfection system impaired it (Figure 5C).

### 3.6 NPAS2 Promoted the Proliferation and Migration of HDFs via CDC25A

**FIGURE 6:**
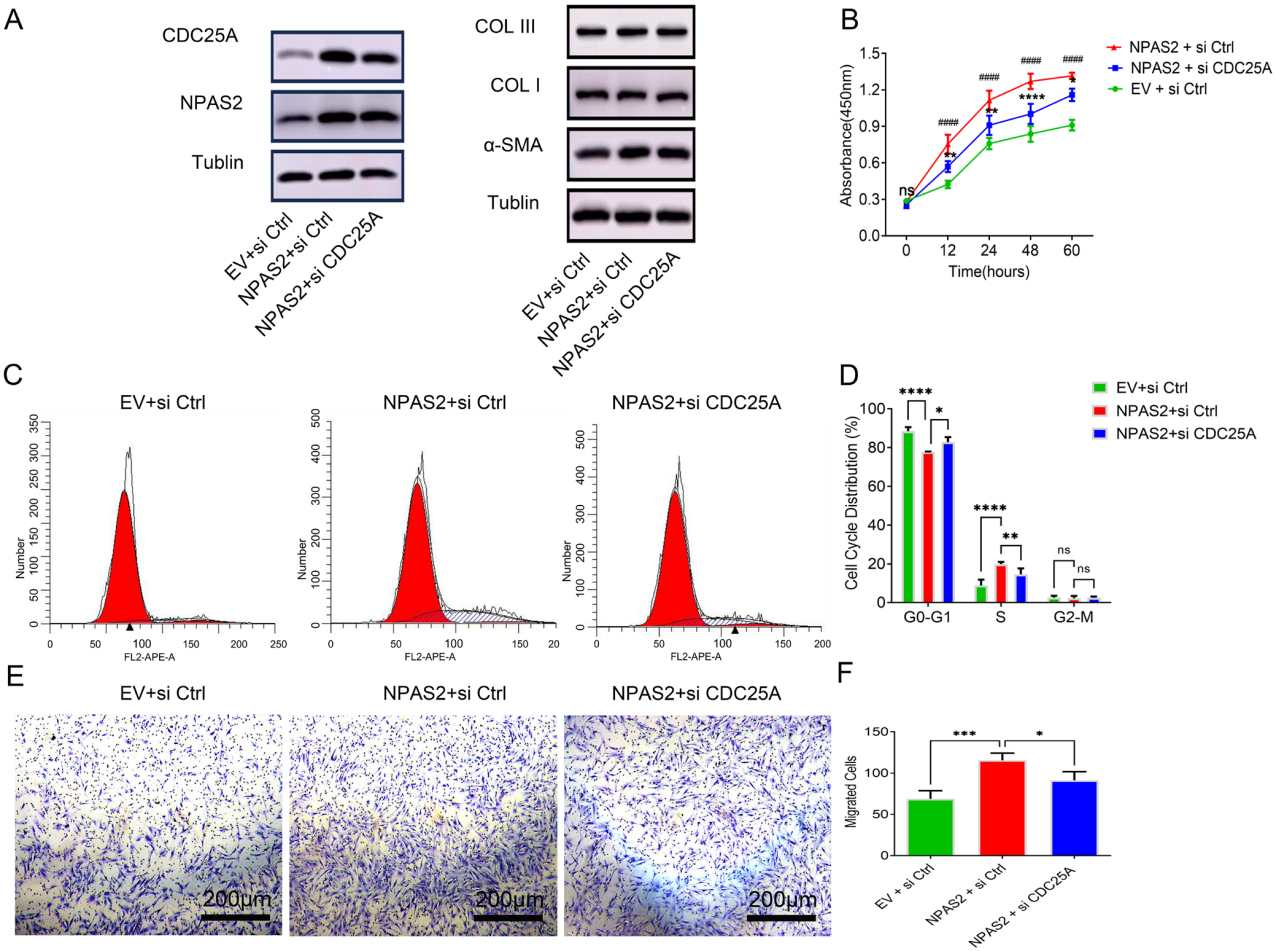
NPAS2 promoted the proliferation and migration of HDFs via CDC25A. (A) Protein expression levels of CDC25A, NPAS2, α‐SMA, COL I, COL III were detected in different cotransfection systems. (B) Proloferative activity was evaluated by CCK8 assay for cotransfected HDFs. (C, D) Flow cytometry was performed to identify the cell cycle distribution for cotransfected HDFs. (E, F) The migratory ability of cotransfected HDFs was detected by transwell assay. EV + si Ctrl represents HDFs cotransfected with control vector and control si RNA; NPAS2 + si Ctrl represents HDFs cotransfected lentiviral vector overexpressing NPAS2 and control si RNA; NPAS2 + si CDC25A represents HDFs cotransfected with lentiviral vector overexpressing NPAS2 and si RNA knocking down CDC25A. Data shown are the mean ± SD. Scale bar, 200 μm. ns, not significant; *p < 0.05; **p < 0.01; ***p < 0.001; ****p < 0.0001.

Furthermore, we successfully transfected lentiviral vectors with negative control or NPAS2 and siCtrl or siCDC25A into HDFs. It did not influence the protein levels of α‐SMA, COL I and COL III in HDFs (Figure 6A). However, both CCK‐8, flow cytometry and transwell assay demonstrated that NPAS2 greatly affected their proliferation and migration, and this effect can be weakened by CDC25A (Figure 6B–F).

### 3.7 NPAS2 Knock‐Down in Rat Tail Wound Inhibited HTS Formation

**FIGURE 7:**
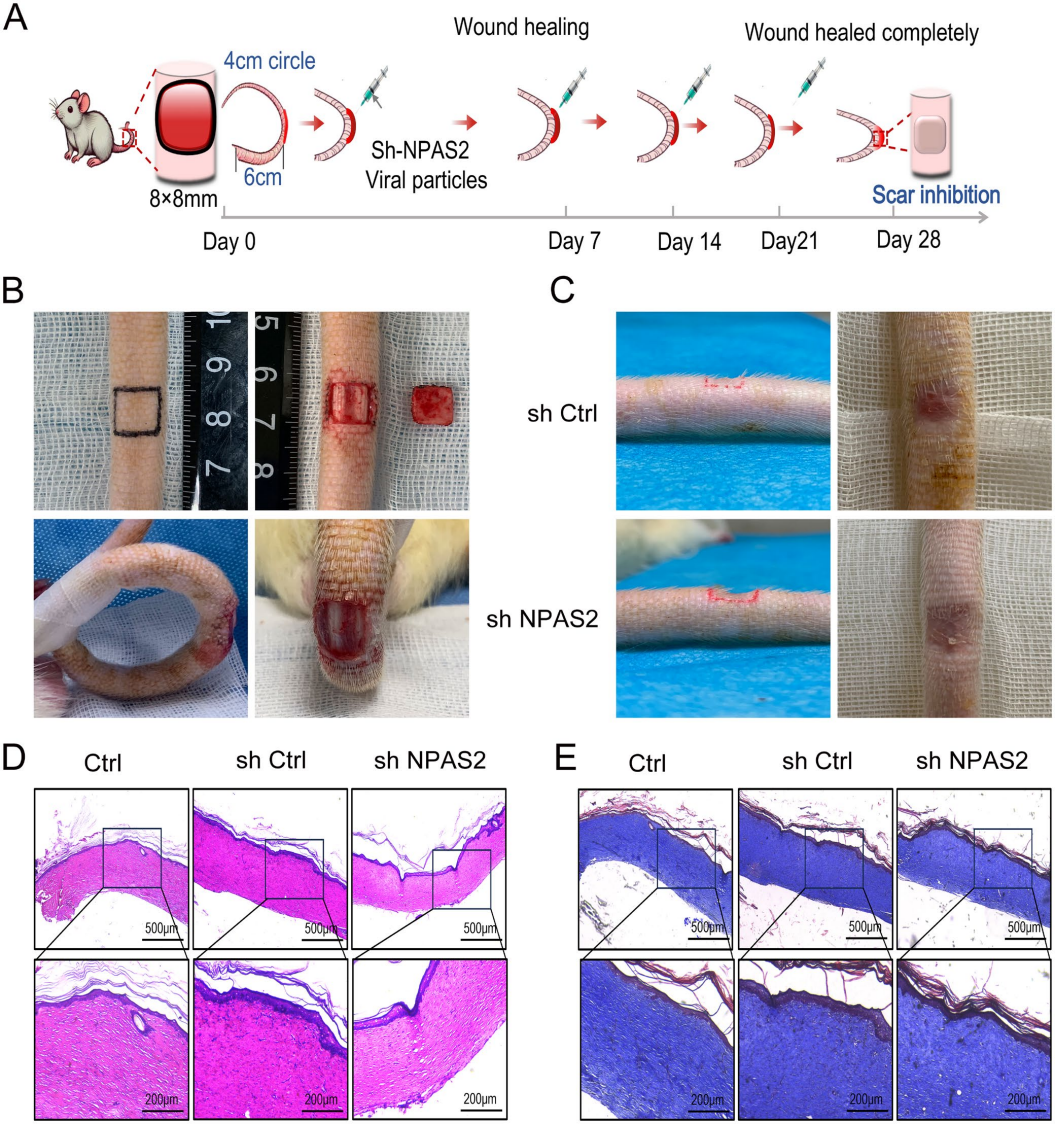
NPAS2 knocked down in rat tail wound inhibited scar formation. (A, B) Schematic illustration of moulding and treatment of rat tail scar. (C) Representative images of rat tail wound healed models. (D, E) H&E staining and Masson's trichrome staining of rat tail wounds. Ctrl, normal SD tail tissues; sh‐Ctrl, SD tail wounds interfered with control lentiviral vector; sh‐NPAS2, SD tail wounds interfered with lentiviral vector knocking out NPAS2. Scale bar, 500 and 200 μm.

SD rats were randomised into two groups: r‐plenR‐sh‐NPAS2 and r‐plenR‐NPAS2‐NC. A full‐thickness 8 × 8 mm square wound was excised on the tail, 6 cm distal to the root, and the tail was secured into a 4 cm‐diameter loop. Viral particles were injected subcutaneously at the midpoint of each wound edge weekly. Figure 7A,B illustrates the experimental schematic and outcomes of tail wound treatment. From the perspective of healing time, there was no significant difference between LV‐NPAS2‐RNAi‐NC and LV‐NPAS2‐RNAi. Both of them obtained a complete re‐epithelialisation on the 25–28th day. However, the general view of them was different. As for the LV‐NPAS2‐RNAi‐NC, the area of the re‐epithelialised region reduced by 25%–35% compared with that of the original wound. Furthermore, the re‐epithelialised tissues with bright red colour and hard texture protruded from the normal skin surface 0.2–0.4 mm. In contrast, the re‐epithelialised regions of LV‐NPAS2‐RNAi were pink, flat and as large as the initial wound, almost with no shrinkage (Figure 7C). In addition, the H&E or Masson's trichrome staining analyses demonstrated that the regenerated dermal tissue of LV‐NPAS2‐RNAi‐NC was thick and held a large number of cells and collagen volume. More importantly, the collagen distribution was dense and irregular. Whereas, that of LV‐NPAS2‐RNAi was similar to normal skin and possessed loose and regular collagen arrangement which was parallel to the epidermis (Figure 7D,E).

## 4 Discussion

The prevalence of HTS is up to 91% following severe burning [32]. Numerous therapeutic strategies have been tried for prevention and treatment of HTS, but no therapy has been universally accepted as the gold standard for all patients. No universal consensus on treatment regimens has been established, and there is limited evidence‐based literature to guide the management. Therefore, the management of HTS remains an ongoing challenge to patients and clinicians.

Darker phototypes and individuals of African and Asian ancestry are known to be susceptible to scarring. Genetic variants increased the risk of scarring formation in these populations, with approximately 5%–10% being familial cases [33, 34, 35]. Therefore, in addition to the tension, depth, and over‐reactive inflammatory response of the wounds and the age of the patient, genetic variation and mutations have been considered to be a risk for the formation of HTS [33]. As a novel therapeutic method, gene therapy can solve the diseases etiologically and has shown great potential in the treatment of refractory diseases. In addition to introducing normal DNA into cells, gene therapy also includes gene correction therapy, gene suppression therapy, gene regulation therapy and other methods. Interestingly, gene therapy can act as a supplement to traditional treatments to reduce or eliminate HTS formation [36], including gene suppression therapy [37, 38] and gene regulation therapy [39, 40].

Circadian rhythm was governed by a complete clock network. Increasingly, studies demonstrated that circadian rhythm disorders caused by unusual clock genes were associated with numerous diseases. As previous studies reported that NPAS2 acts as a tumour suppressor in colorectal and breast cancers [41, 42]. However, Yuan et al. [43] confirmed that NPAS2 played a tumour‐promoting gene in hepatocellular carcinoma (HCC) and upregulated NPAS2 was associated with aggressive clinical problems and poor prognosis, which was consistent with its role in acute myeloid leukaemia [44]. The mixed results may be due to cellular heterogeneity. A recent study demonstrated that NPAS2^−/−^ mice showed a faster wound healing process and larger collagen deposition than NPAS2^+/−^ and WT groups. Meanwhile, fibroblasts isolated from NPAS2^−/−^ mice displayed strong proliferation, migration, and contraction forces [27]. However, our study confirmed that the expression of CLOCK in HTS‐Fs was significantly lower than that in HDF at both mRNA and protein levels, while NPAS2 was significantly upregulated (Figures 1 and 2). Therefore, this shift in expression pattern may lead to the replacement of the CLOCK‐BMAL1 complex by the NPAS2‐BMAL1 complex under pathological conditions, making it a key transcription factor regulating fibroblast function. Moreover, NPAS2 overexpressed in HDFs promoted its survival, migration and collagen deposition (Figure 3). These contradictions might be attributed to species specificity and other unknown mechanisms.

In addition to wound healing and tumorigenesis, dysregulation of NPAS2 is associated with the occurrence of pulmonary fibrosis [45], hepatic fibrosis [28] and atrial fibrosis [46]. HTS, considered to be fibrosis of the skin, arises from traumatic or surgical injuries and the subsequent aberrant form of wound healing, which is characterised by enhanced proliferation, migration activity, and secretion. In this study, we introduced or silenced NPAS2 in HDFs or HTS‐Fs, respectively, to explore the influence of NPAS2 on fibroblasts. The results showed that overexpression of NPAS2 in HDFs can up‐regulate the proliferation, migration activity and secretion of HDFs, while knockdown of NPAS2 in HTS‐Fs negatively regulates that of HTS‐Fs. Such results suggested that NPAS2 was conducive to the transformation of fibroblasts into myofibroblasts, which was consistent with that of NPAS2 in other tissue fibrosis.

Cell division cycle factor 25A (CDC25A), a critical cell cycle regulatory protein, has specific phosphatase activity. It activates cyclin‐dependent protein kinase (CDK) by dephosphorylation and thus promotes cell proliferation via driving G0*–*G1 to S [47]. Numerous studies demonstrated that CDC25A influenced cell mitosis, proliferation as well as the development and poor prognosis of malignant tumours [48, 49, 50]. Meanwhile, previous studies speculated that CDC25A was one of the direct transcriptional regulatory target genes of NPAS2, which was confirmed in breast cancer and hepatocellular carcinoma [43, 51]. In this study, we demonstrated that NPAS2 transcriptionally activated CDC25A through the heterodimer of NPAS2 and BMAL1 direct binding to an E‐like box of the CDC25A promoter, which was consistent with previous studies (Figure 5). When CDC25A was silenced, the function of NPAS2 in promoting the proliferation and migration of HDFs was suppressed (Figure 6). Therefore, CDC25 is a target for NPAS2 to regulate the proliferation and migration in HDFs.

The rabbit ear model has been widely used to study HTS formation. However, due to the low thickness of the ventral side of the rabbit ear, the perichondrium needs to be treated with extreme caution to avoid damaging the underlying cartilage, which may increase the rates of surgical failure [52, 53]. Meanwhile, some attempts have been made to develop a xenograft model of HTS by grafting human HTS onto nude mice [54]. However, the lack of an immune response hinders the study of therapeutic molecules in this model [55]. Recently, several groups have also attempted to develop rodent models for HTS. These murine models are inexpensive and easy to handle, but their wound healing patterns differ from those of humans due to the rapid contraction of the panniculus carnosus muscles [56]. To mitigate the effect of rapid wound contraction, mechanically rival forces acting on wounds have been attempted. In this model, increased mechanical tension and blocked wound re‐epithelialization amplify the function of myoblasts, ultimately leading to HTS‐like features that are similar to human HTS [57]. In this study, we successfully constructed scar models in the rat tails as described previously [58]. The panniculus carnosus muscles of rat tails are weak and easy to handle, and the mechanical stretch induced by the ring fixation of the rat tail can overcome the wound contraction, thus simulating the microenvironment prone to HTS formation and leading to HTS‐like features that are similar to human HTS.

In order to explore the intervention of NPAS2 on HTS, we constructed adenovirus vectors carrying murine NPAS2 and applied them to rat tail scar models. As shown in Figure 7C, the rat tail wounds with NPAS2 knocking down exhibited better appearances with less contraction and collagen deposition. Moreover, in the LV‐NPAS2‐RNAi group, the lateral view surface of re‐epithelialized skin was lower than that of the peripheral normal skin surface, which might be owing to the fact that the time we observed was limited and only until the wound healed. Generally, the regeneration of subcutaneous fat tissue will take a much longer time. Therefore, we will continue to pay attention to the development of HTS. It is not perfect yet, but our experiment initially confirmed that the knocking down of NPAS2 could improve the scar healing process and perform as a target for scar treatment.

Therefore, although this study has some limittions, it elucidated the potential mechanisms by which NPAS2 influences the HTS formation, and confirmed that NPAS2 and its gene modifications are promising targets for HTS management.

## 5 Conclusions

**FIGURE 8:**
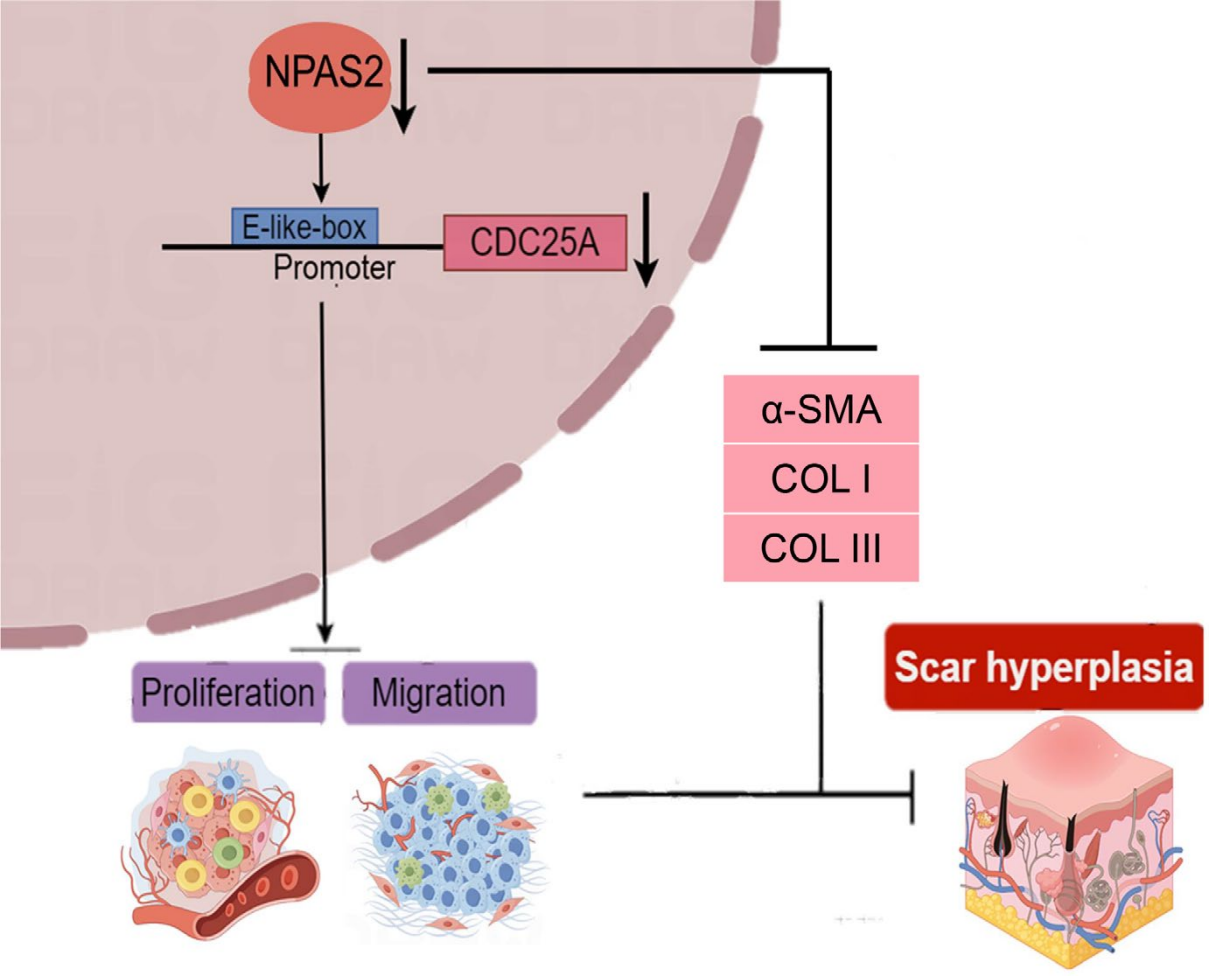
The mechanism diagram of NPAS2 inhibiting HTS. NPAS2 restrained the cell proliferation and migration, and decreased the expression of ECM.

In summary, As shown in Figure 8, NPAS2 promoted the activation of HDFs, which is characterised by stronger proliferation and migration and the higher expression level of α‐SMA, COL I and COL III. In this, the proliferation and migration effects of NPAS2 were mediated by CDC25A. Furthermore, NPAS2 knocked down in rat tail wounds inhibited the HTS formation. Therefore, NPAS2 may serve as a potential therapeutic target for HTS in the future

## Author Contributions

**Pei Wei:** conceptualization (equal), data curation (equal), investigation (equal), methodology (equal), validation (equal), writing – original draft (equal), writing – review and editing (equal). **Yongqiang Xiao:** conceptualization (equal), data curation (equal), visualization (equal). **Zhaorong Xu:** project administration (equal), resources (equal), supervision (equal). **Xiaodong Chen:** investigation (equal), resources (equal), software (equal). **Qiong Jiang:** formal analysis (equal), methodology (equal), validation (equal), visualization (equal). **Yu Fu:** investigation (equal), methodology (equal), software (equal). **Jianji Yan:** methodology (equal), resources (equal), software (equal). **Zhaohong Chen:** funding acquisition (equal), project administration (equal), supervision (equal), validation (equal), visualization (equal). **Pengfei Luo:** data curation (equal), project administration (equal), supervision (equal), validation (equal), visualization (equal). **Huazhen Liu:** conceptualization (equal), formal analysis (equal), investigation (equal), supervision (equal), writing – review and editing (equal).

## Ethics Statement

The authors have nothing to report.Our study were approved by Fujian Medical University Union Hospital (2021KJCX081).

## Consent

The authors have nothing to report.

## Conflicts of Interest

The authors declare no conflicts of interest.

## Data Availability

The data that support the findings of this study are available fromthe corresponding author upon reasonable request.
